# Development of a murine model to study the cerebral pathogenesis of *Aspergillus fumigatus*

**DOI:** 10.1128/msphere.00468-23

**Published:** 2023-11-27

**Authors:** Martin T. Kelty, Sarah R. Beattie

**Affiliations:** 1 Department of Pediatrics, Carver College of Medicine, University of Iowa, Iowa City, Iowa, USA; University of Georgia, Athens, Georgia, USA

**Keywords:** fungal infection, central nervous system infections, *Aspergillus*

## Abstract

Fungal infections of the central nervous system are associated with high mortality and limited treatment options due to the poor permeability of the blood brain barrier to most antifungal drugs. The most common pathogenic mold is *Aspergillus fumigatus*, which typically causes invasive pulmonary disease that can disseminate to extrapulmonary organs, including the brain, resulting in cerebral aspergillosis (CA). Currently, the best treatment option for CA is antifungal therapy combined with resection of infected tissue; however, mortality rates of these infections still approach 100%. Despite these unacceptably high mortality rates, relatively little is known about the fungal determinants that allow *Aspergillus* to successfully establish infection and grow within the brain. Here, we present a murine model designed to study the fungal pathogenesis of CA using tail vein inoculation in C5-complement-deficient mice. In this model, mice develop a robust fungal burden in the brain and display hallmarks of invasive aspergillosis seen in both murine models of invasive aspergillosis and in human cases of CA. We highlight the role of one fungal transcription factor, PacC, in the hematogenous dissemination of *A. fumigatus* to extrapulmonary organs including the brain. This model will enable pathogenesis studies in a largely unexplored area of *A. fumigatus* infections with the goal of identifying novel targets and pathways for the development of more efficacious antifungal therapies to treat these infections.

**IMPORTANCE:**

Molds are environmental fungi that can cause disease in immunocompromised individuals. The most common pathogenic mold is *Aspergillus fumigatus,* which is typically inhaled into the lungs and causes invasive pulmonary disease. In a subset of these patients, this infection can spread from the lungs to other organs including the brain, resulting in cerebral aspergillosis. How *A. fumigatus* causes brain disease is not well understood and these infections are associated with extremely high mortality rates. Thus, we developed an animal model to study the pathogenesis of cerebral aspergillosis to better understand this disease and develop better treatments for these life-threatening infections.

## INTRODUCTION

Mold infections are a growing concern among susceptible populations including solid organ and hematological stem cell transplant recipients, chemotherapy recipients, people living with HIV/AIDS, and other cohorts of immunocompromised individuals (1). Additionally, continued advancements in medical therapies (e.g., new anti-cancer immunotherapies [2]) and emerging co-morbidities (e.g., COVID-19 [(3, 4]) are creating new susceptible populations to these devastating infections. The most common pathogenic mold is *Aspergillus fumigatus*, an environmental pathogen that is found worldwide in both indoor and outdoor environments. Constant exposure to airborne fungal conidia by inhalation has resulted in efficient anti-*Aspergillus* immunity in the lungs of healthy individuals where conidia are cleared by resident alveolar macrophages before any pathology occurs. However, when immune defenses are dampened, *Aspergillus* conidia can germinate and penetrate within the lung tissue and establish invasive pulmonary aspergillosis (IPA), the most common form of invasive aspergillosis (5).

The growth of *Aspergillus* within the lungs is associated with tissue destruction and angioinvasion, granting fungi access to the bloodstream where they can disseminate to extrapulmonary sites. Dissemination occurs in ~50% of cases of invasive aspergillosis, and within these patients, ~20%–40% are estimated to develop cerebral aspergillosis (CA), or *Aspergillus* infection in the brain (5). In addition to dissemination from the lungs, which accounts for the majority of cases, CA can also occur secondary to *Aspergillus* sinusitis or from complications following surgery (6). CA is characterized by fungal growth, necrosis, and neutrophilic infiltration in the brain tissue and is among the most fatal forms of aspergillosis, with mortality rates of 70%–100% (7–9). Currently, the best course of treatment includes both voriconazole, an azole antifungal that can readily cross the blood-brain barrier (BBB), combined with resection of the infected tissue, when possible (10).

Despite the high mortality rates, little is known about the pathogenesis of CA and the fungal factors required to penetrate and grow within the brain tissue. One challenge in studying this disease is the lack of a robust animal model to study the natural route of infection beginning in the lungs and then disseminating to the brain. Though cerebral symptoms have been observed in murine inhalation models of IPA, morbidity and mortality from lung infection occur too rapidly for robust brain infection to occur. Recently, Sullivan et al. (11), developed a “two-hit” model, which uses intranasal inoculation followed by intravenous inoculation to model pulmonary disease with secondary dissemination to the brain. The strength of this model is the establishment of pulmonary infection first to better reflect the immune status of the host during cerebral infection. However, the two routes of inoculation create a complex system where *Aspergillus* can establish CA via hyphal fragments disseminating from the lung and conidia inoculated directly into the bloodstream. To study the fungal pathogenesis of CA and the establishment of brain disease, the dual inoculation route could be a confounding factor and make interpreting results difficult. Thus, we opted for a model that uses a single intravenous route of inoculation for our foundational studies to identify fungal pathways/proteins required to establish CA. These pathways can then be explored in other *in vivo* and *in vitro* models of aspergillosis and host cell interactions to understand the role of fungal genes in general hematogenous dissemination and specific interactions with endothelial cells of the BBB.

Here, we present a model of disseminated aspergillosis to study the establishment of brain infection in mice. We demonstrate that direct inoculation of *A. fumigatus* conidia into the lateral tail vein of C5-complement-deficient mice results in disseminated disease with robust fungal growth in the brain. These animals have clinical symptoms of central nervous system (CNS) disease, and the histological findings replicate features seen in human infections. Using NanoString nCounter technology, we evaluated the murine immune response to *A. fumigatus* in the brain and observed immune signatures consistent with known immunological responses to aspergillosis. Finally, we tested the role of the *A. fumigatus* transcription factor PacC, a known regulator of host epithelial cell invasion, in CA and observed an inability of the Δ*pacC* mutant strain to effectively establish disseminated disease, including brain infection. The goal of this work is to develop a model to begin unraveling the fungal biology required to establish CA and to identify potential targets for the improved treatment of these life-threatening infections.

## RESULTS

### Corticosteroid-treated mice are susceptible to disseminated aspergillosis

The natural route of *A. fumigatus* infection usually occurs through inhalation of conidia resulting in a pulmonary infection from which hyphal fragments can invade blood vessels and disseminate to extrapulmonary sites. In murine models of IPA, pulmonary disease induces rapid onset of morbidity and mortality. Though we have been able to identify culture-positive brain samples from these animals, the fungal burden is too low to study the development of cerebral infections (Fig. S1A). Thus, to develop a clinically relevant model of cerebral aspergillosis, we moved to an intravenous inoculation strategy to establish robust brain infection. Previously established disseminated models of aspergillosis have demonstrated that tail vein inoculation results in high fungal burden in the brains of mice including complement-deficient DBA/2J mice (12), immunocompromised outbred mice (13), and immunocompetent inbred mice (14). Similarly, we were able to detect *A. fumigatus* in the brains of immunocompetent outbred CD-1 mice inoculated intravenously. However, the fungal burden was only detectable in 3/5 mice (Fig. S1B), and at the time of sacrifice, no animals displayed symptoms of aspergillosis or CNS disease. These results suggest that immunosuppression is required to establish disseminated disease in outbred mice.

**Fig 1:**
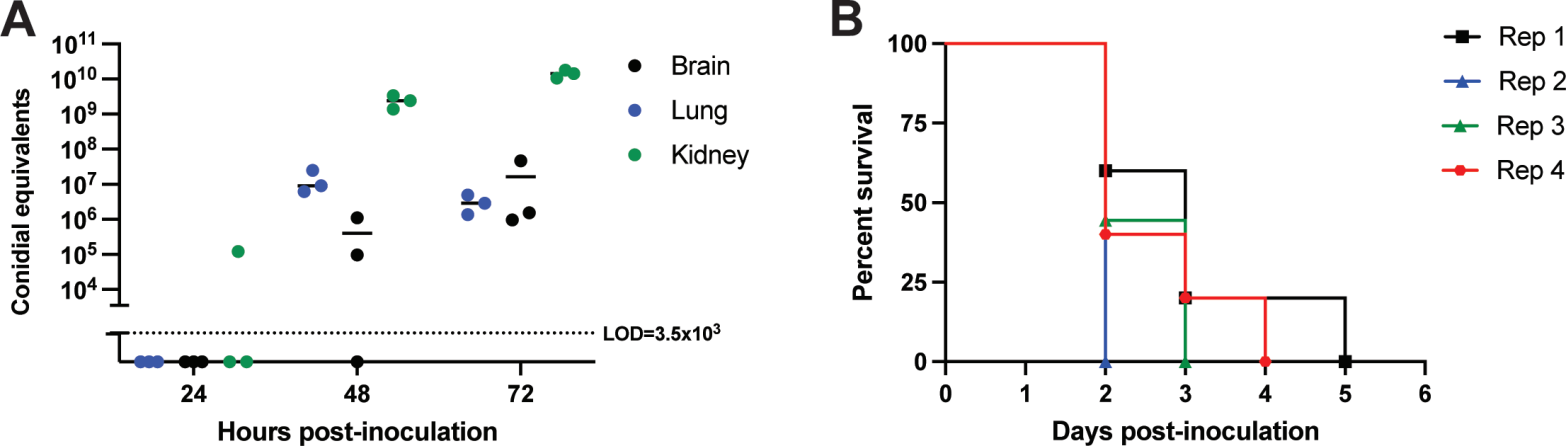
Steroid treatment renders outbred mice susceptible to disseminated aspergillosis. (**A**) Fungal burden of CD-1 mice treated with 40 mg triamcinolone/kg of body weight and inoculated with 1 × 10^5^ *A. fumigatus* CEA10 via lateral tail vein. Dotted line represents the limit of detection (LOD) for fungal burden analyses. Data are plotted as individual values with mean. *n* = 3 mice/group. (**B**) Survival analysis of four individual replicates inoculated as described in panel A. *n* = 5 mice per rep. except 3, where *n* = 9.

The most common models of invasive pulmonary aspergillosis require the use of immunosuppressive drugs, such as corticosteroids or chemotherapy agents, to render mice susceptible to the disease (15). Thus, we treated mice with a single high dose (40 mg/kg of body weight) of triamcinolone 24 hours prior to inoculation, then inoculated them with 1 × 10^5^ conidia of *A. fumigatus* reference strain, CEA10, via the lateral tail vein. Using a previously established quantitative PCR method (12), we measured the fungal burden of lungs, kidneys, and brains at 24, 48, and 72 hours post inoculation (hpi). While no organism was detectable at 24 hpi except in the kidneys of 1/3 mice, by 48 hours all mice had high kidney and lung burden (~10^8^ conidial equivalents [CE] and ~10^7^ CE, respectively) while 2/3 mice had detectable brain burden (Fig. 1A). At 72 hpi, all mice had detectable fungal burden in all tested organs with similar burden in the lungs and brain (~10^6^–10^7^ CE) and the highest burden in the kidneys (~5 × 10^8^ CE; Fig. 1A).

An essential step in the development of invasive aspergillosis is the germination of conidia into hyphae. Since our assay to measure fungal burden relies on the detection of fungal DNA, it is possible that the fungal DNA detected in murine organs represents the accumulation of ungerminated conidia and not true invasive growth. Thus, to ensure that our fungal burden results are indicative of hyphal growth *in vivo*, we inoculated immunosuppressed CD-1 mice with an uracil/uridine auxotroph, CEA17, which germinates poorly within the lung (~10% of wild type [WT]) and is avirulent in pulmonary models of invasive aspergillosis (16). In agreement with previous studies, CEA17 was undetectable by qRT-PCR in the lungs of animals at 72 hpi suggesting that the growth defect of this strain is not affected by alternate routes of inoculation (Fig. S2). Similarly, CEA17 was not detectable in the kidneys or brains of mice, indicating that uracil and uridine biosynthesis is required for growth in extrapulmonary organs. Additionally, this result provides confidence that the fungal burden detected in this model is the result of germination and invasive growth and not the detection of the inoculum accumulating in organs of interest.

In this model, mice developed symptoms within 48 hours and most succumbed to infection by day 3 or 4 post-inoculation (Fig. 1B). Observed symptoms included lethargy, inability to walk/splayed hind legs, respiratory distress, and hematuria; we did not observe any symptoms specific to CNS disease such as head tilting/loss of equilibrium, convulsions, or tremors. In addition, while developing this model, we observed experiment-to-experiment variability in mortality with some experiments resulting in 100% mortality by day 2 (Fig. 1B). The brain burden is also highly variable, with coefficients of variation (%CV) of 153 and 160 at 48 and 72 hours respectively, compared to %CV = 30 and 11 for kidneys and %CV = 76 and 58 for lungs at 48 and 72 hours, respectively. Finally, the lack of CNS symptoms, high fungal load in the kidney, and hematuria suggests that morbidity and mortality in these animals are driven by overwhelming fungal growth in the kidney. Together, these data suggest that this model is not reproducible enough to study the pathogenesis of CA; however, we were successful in establishing fungal burden in the brain via lateral tail vein in immunosuppressed outbred mice.

### *A. fumigatus* establishes reproducible CA in C5-complement-deficient mice

Studying disseminated aspergillosis in the context of corticosteroid use is clinically relevant, given that 25%–50% of patients with CA are being treated with corticosteroids for hematological malignancies, graft versus host disease, or transplant therapy (17, 18). However, we found that corticosteroid-treated mice were highly susceptible to intravenous challenge with *A. fumigatus* but interexperimental variability made this model untenable to evaluate the pathogenesis of CA. The use of corticosteroids has pleiotropic effects on the host including changes in metabolism, immune, endothelial, and epithelial cell functions, all of which may confound studies of endothelial cell interactions (19, 20). Therefore, we chose a model well-known for its utility in studying fungal meningitis: the C5-complement-deficient mouse strain A/J. These mice have been used extensively to study cryptococcal meningitis caused by yeast of the *Cryptococcus* genus (21). The C5-deficiency results in the loss of the activation of the complement cascade and delayed neutrophil recruitment to the site of infection leaving these mice susceptible to a myriad of bacterial and fungal infections (22–25). Similar C5-complement-deficient mouse strains including DBA/2 and S/J mice have been used in disseminated models of aspergillosis with robust brain burden; however, to our knowledge, these models have primarily been used for antifungal efficacy studies and have not been validated for or employed to elucidate the mechanism of fungal dissemination and growth in the brain (12, 26, 27).

To determine whether A/J mice can be used to model CA, we first tested whether intranasal instillation of conidia would result in the robust establishment of brain burden from pulmonary dissemination. However, like immunosuppressed CD-1 mice, this inoculation route fails to produce disseminated infection in the brain after 6 and 14 days (data not shown). Consistent with previous studies that report A/J mice are not susceptible to IPA without additional immunosuppression (26, 27), these mice did not develop symptoms of invasive aspergillosis and only 1/4 animals had a detectable burden in the lungs at 6 days post-inoculation (data not shown).

**Fig 2:**
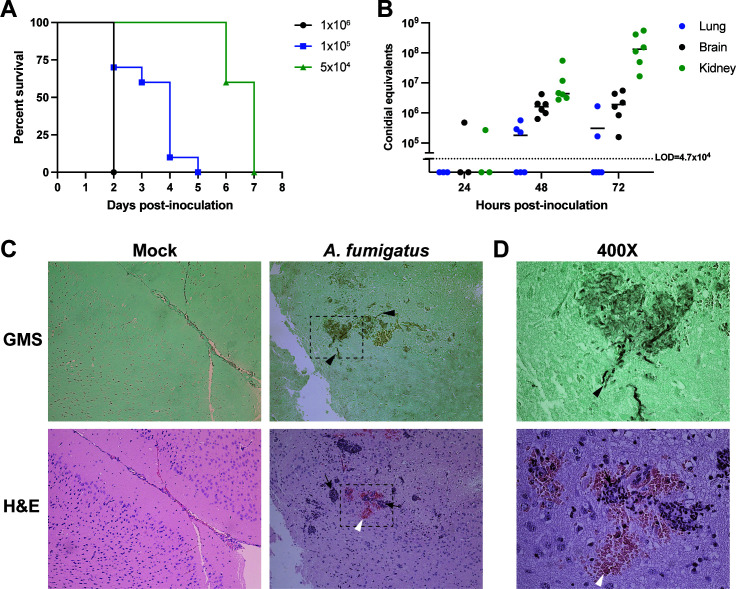
C5-complement-deficient mice are susceptible to cerebral aspergillosis. (**A**) Survival analysis of A/J mice inoculated with 1 × 10^6^ (*n* = 8 mice), 1 × 10^5^ (*n* = 10 mice from two independent replicates), and 5 × 10^4^ (*n* = 5 mice) *A. fumigatus* CEA10 via lateral tail vein. (**B**) Fungal burden of A/J mice inoculated with 1 × 10^5^ CEA10 conidia at 24 (*n* = 3 mice), 48 (*n* = 6 mice from two independent experiments), and 72 (*n* = 6 mice from two independent experiments) hours post-inoculation. Dotted line represents the limit of detection (LOD). Data are plotted as individual values with the mean. (**C**) Hematoxylin and eosin (H&E) and Grogott’s methanamine silver (GMS) staining of coronal sections of brains harvested from A/J mice 72 hours post-inoculation with 1 × 10^5^ CEA10 or PBS (mock) via lateral tail vein at 100×. Black arrowheads highlight examples of hyphae within a fungal lesion in the cortex. Black arrows show areas of inflammatory cell infiltrate and white arrowheads highlight regions of hemorrhage. Dashed box shows the magnified region in panel D at 400× magnification. Representative images from *n* = 2 mice.

Next, we performed survival analyses with A/J mice inoculated via lateral tail vein with a range of inoculum concentrations. We observed a correlation between inoculum density and rate of mortality; mice inoculated with 1 × 10^6^ CEA10 conidia began to show symptoms within 36 hours and all succumbed by 48 hours (Fig. 2A). All five of these animals showed CNS-specific symptoms including a head tilt and spinning behavior. Mice inoculated with 5 × 10^4^ CEA10 conidia did not begin to show symptoms until 4 days post-inoculation and succumbed to infection by day 7. However, in these mice, we did not observe any CNS-specific symptoms, and fungal burden analysis on brains from mice at the time of euthanasia revealed that only one of five animals had detectable *A. fumigatus* in the brain with high burden in the kidney (Fig. S3). These results suggest at the lowest tested dose, though A/J mice are susceptible to intravenous *A. fumigatus*, the inoculum is either too low to establish a detectable burden in the brain or the dose is low enough that the animals can clear the brain infection and likely succumb to the effects of high kidney burden.

At the intermediate dose of 1 × 10^5^, we observed the onset of symptoms at 48 hpi and most mice reached endpoint criteria by 4 days post-inoculation (Fig. 2A). At this dose, ~50% of mice showed CNS-specific symptoms and all mice showed non-specific symptoms of invasive aspergillosis such as lethargy, inability to walk, and hunched/ruffled posture. Importantly, compared to steroid-treated mice, we observed less variability in mortality. Though some animals did succumb to infection by 48 hpi in some experiments, there were no instances where all animals reached mortality within this time, allowing us to collect sufficient samples for 48- and 72-hour timepoints in subsequent experiments. We tested the kinetics of fungal growth and dissemination and observed that, like the corticosteroid model, at 24 hpi, only 1/3 of mice had detectable burden in the brain and kidneys. However, by 48 and 72 hpi, all mice had detectable fungal burden in the kidneys and brains (Fig. 2B). In contrast to corticosteroid-treated CD-1 mice, we observed that the lungs were the most variable in terms of fungal burden with several mice just at the limit of detection. Moreover, the brain has the most consistent burden with %CV = 69 and 85 at 48 and 72 hours, respectively, compared to %CV = 151 and 100 and %CV = 127 and 219 for kidney and lungs at each respective timepoint. Coronal sections of brains prepared from mice 72 hpi showed fungal lesions in the cortex with some growth extending through the corpus callosum. Lesions were characterized by the presence of fungal hyphae, areas of hemorrhage, and cellular infiltration (Fig. 2C and D). These histological findings are consistent with lesions seen in humans (6, 28). Together, these data demonstrate that C5-complement-deficient mice are susceptible to CA with characteristics observed in human disease.

### Neuroimmune transcriptional profiling shows hallmarks of invasive aspergillosis

**Fig 3:**
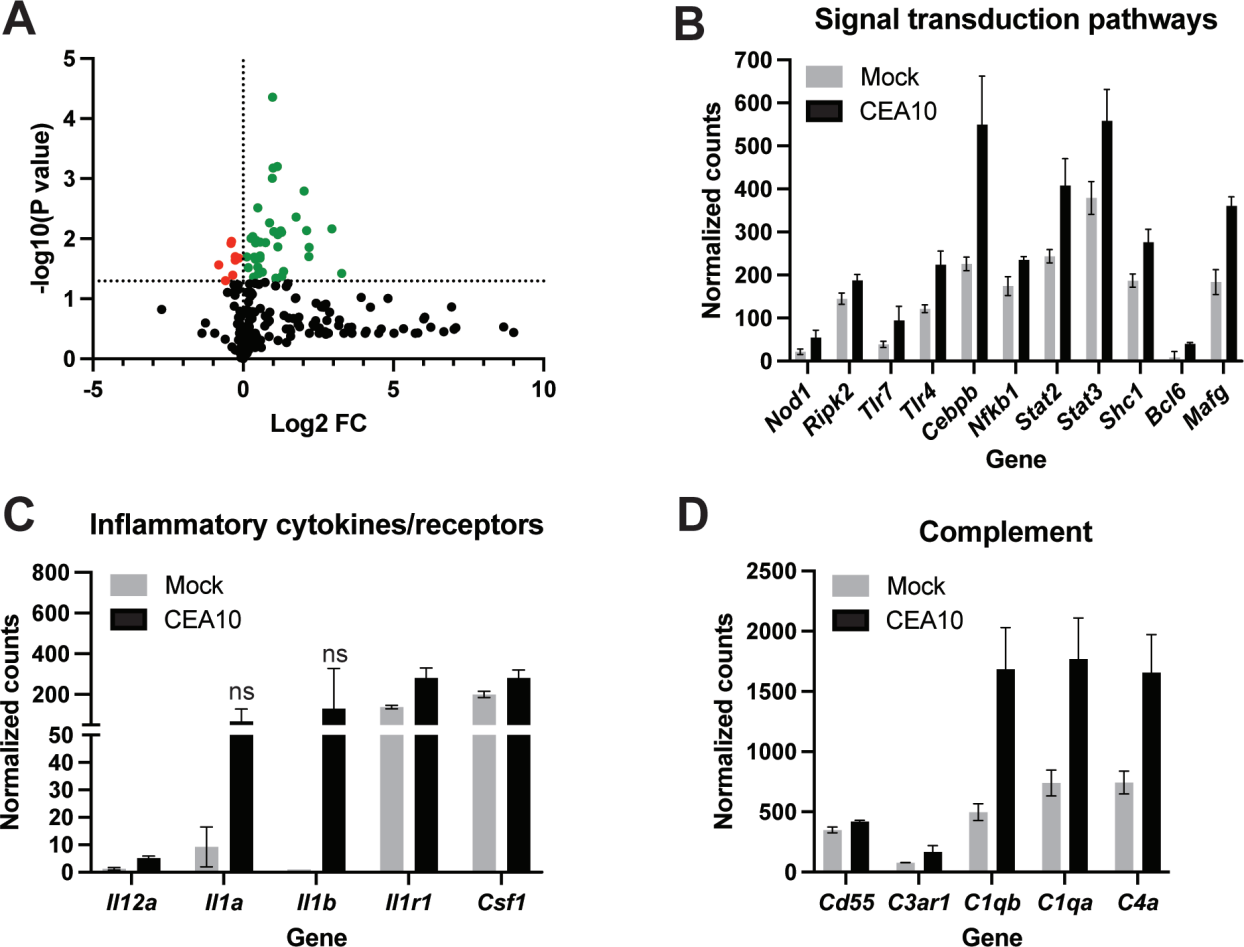
NanoString profiling of brain tissue reveals immunological signatures of invasive aspergillosis. (**A**) Volcano plot of NanoString analysis with significantly reduced (red) and increased (green) genes. (**B–D**) Individual NanoString expression of signal transduction pathways (**B**), inflammatory cytokines/receptors (**C**), and complement components (**D**) from brains of mice inoculated with PBS (mock, gray bars) or 1 × 10^5^ CEA10 conidia (black bars) by lateral tail vein. Data represent the mean and SD of three mice per group. All genes are significant compared to mock (*P* < 0.05 by unpaired, two-tailed *t*-test) unless denoted as not significant (ns).

The immune response to *A. fumigatus* in pulmonary infections has been well-characterized and includes several signaling pathways that regulate proinflammatory responses. To determine the immune profile of cerebral aspergillosis in our complement-deficient mouse model, we performed NanoString analysis of inflammatory gene expression on brains harvested from mice 72 hpi with *A. fumigatus* strain CEA10. This analysis revealed 45 genes that were significantly different (*P* < 0.05 by unpaired, two-tailed *t*-test; Table 1; Table S1) from vehicle-treated controls. Of these, eight genes were significantly reduced, while 37 genes were significantly increased in response to *A. fumigatus* challenge (Fig. 3A; Table 1).

**TABLE 1:** Significantly (*P* < 0.05) changed genes from NanoString expression profiling of mouse brains inoculated with PBS (mock) or *A. fumigatus* via lateral tail vein^*a*^

Gene	CEA10 avg	Mock avg	Fold change	*P* value
Cfl1	13,284.18	17,623.74	0.753766	0.011969
Tgfbr1	1,109.529	1,455.222	0.762447	0.01108
Hmgb1	5,751.937	6,884.18	0.83553	0.022811
Cdc42	36,005.86	43,087.05	0.835654	0.019889
Gnb1	17,804.93	19,680.84	0.904684	0.0211
Grb2	1,454.827	1,340.354	1.085405	0.020089
Cd55	419.2797	349.7827	1.198686	0.009887
Map3k1	158.0224	126.3795	1.25038	0.009215
Ripk2	187.2647	145.0882	1.290695	0.020214
Mapkapk2	499.0479	376.0065	1.327232	0.021921
Tradd	64.34246	48.42729	1.328641	0.011726
Nfkb1	235.3807	174.5492	1.348507	0.010676
Nfe2l2	521.9612	371.3346	1.405636	0.00306
Stat3	558.6536	379.2716	1.472964	0.019385
Shc1	275.917	187.1731	1.474128	0.01137
Tyrobp	2,122.422	1,439.222	1.474701	0.021007
Stat2	407.8744	243.8229	1.67283	0.011614
Tlr4	224.31	121.7513	1.842362	0.005441
Mafg	361.0439	183.9049	1.96321	0.000986
Flt1	105.2722	53.29976	1.975098	4.39E-05
Elk1	179.8305	89.99189	1.998297	0.000662
Il1r1	281.1891	138.1878	2.034833	0.007629
Mef2a	679.4803	309.4013	2.196113	0.000625
Hspb1	166.7653	75.07879	2.221204	0.013653
C4a	1,657.707	743.3999	2.229899	0.008545
C1qa	1,770.321	740.0884	2.392039	0.00741
Cebpb	549.7566	226.0968	2.43151	0.007818
C1qb	1,684.441	497.6322	3.384912	0.004352
Il12a	5.125667	1.257926	4.074697	0.00161
Masp2	47.56982	11.01472	4.318751	0.007343
Bcl6	39.85593	8.730147	4.565322	0.019898
Ifi27l2a	664.3088	144.5814	4.594703	0.013954
Ltb4r1	7.756624	1	7.756624	0.00687
Ccl17	96.78424	169.914	0.569607	0.027227
Csf1	280.9237	199.9268	1.405133	0.029638
Map3k7	2,317.437	2,056.03	1.127141	0.030332
Nod1	54.98996	21.62204	2.543237	0.034904
Cysltr1	119.0964	76.69956	1.552765	0.036144
Myc	34.89834	3.592506	9.714207	0.037802
Mrc1	315.909	221.3619	1.427116	0.038234
Trem2	93.09856	118.2843	0.787075	0.040506
Tlr7	95.01607	38.97771	2.437703	0.042558
Smad7	294.0783	231.5944	1.269799	0.043433
C3ar1	167.3461	78.70614	2.126214	0.045426
Ptgfr	72.8863	109.6665	0.664618	0.049785

^*a*^ Average of three mice per group. *P* values are calculated using an unpaired, two-tailed *t*-test.

Included in the increased genes are several signal transduction pathways with known roles in anti-*Aspergillus* immunity. We observed upregulation of *Tlr7* and *Tlr4* (29–31), which activate transcription factors including NF-κB (32, 33) and CEBP-β (Fig. 3B) (32, 34). These transcription factors regulate pro-inflammatory gene expression, including IL-1 and IL-12 family cytokines, both of which are important in anti-*Aspergillus* immunity (35–37). Though IL-1α and IL-1β were increased in mice challenged with CEA10, only the IL-1 receptor (*Il1r1*) and *Il12a* were significantly upregulated at 72 hpi (Fig. 3C). In addition, we observed upregulation of *Nod1*, an intracellular receptor previously shown to increase upon *A. fumigatus* exposure (38), and its associated receptor kinase *Rip2*, which also activates NF-κB signaling. We also identified several other transcription factors that have been shown to respond transcriptionally to *A. fumigatus* challenge including *Stat2* (32), *Stat3* (39, 40), the STAT scaffold *Shc1*, *Bcl6* (32), and *cMaf* (Fig. 3B) (41).

Finally, we observed significant increases in the complement pathways including *C1qb*, *C1qa*, *C4a*, *C3ar1*, and the complement inhibitor *Cd55* (Fig. 3D). These results are consistent with previous reports of induction of complement upon exposure to *A. fumigatus* (32, 42, 43). Furthermore, human cerebral spinal fluid from CA patients shows an increase in C1q, C3, C3a, and C5 (44, 45), and people with defects in complement activation or who are taking the anti-C5 monoclonal antibody therapy, eculizumab, are at risk for invasive aspergillosis (46, 47). Together, these results demonstrate that C5-complement-deficient mice develop robust CA and show immunological hallmarks of invasive aspergillosis in the brain in a clinically relevant murine model.

### The transcription factor PacC is required for hematogenous dissemination of *A. fumigatus*

**Fig 4:**
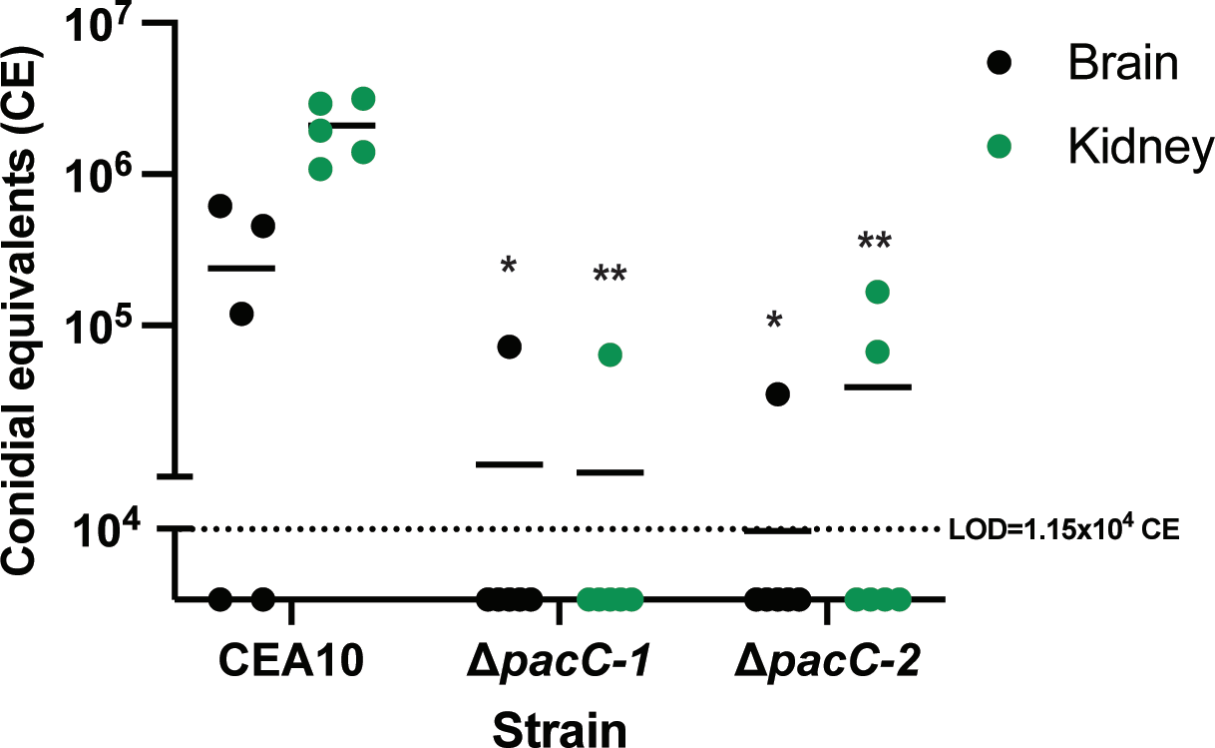
PacC is required for hematogenous dissemination to the brain and kidneys. Fungal burden of mice inoculated with CEA10 or Δ*pacC-*deletion mutants in the brain and kidneys collected 48 hours post-inoculation. *n* = 5 mice for CEA10, and 6 mice for Δ*pacC-1* and Δ*pacC-2*. Dotted line represents the limit of detection (LOD) for fungal burden analyses. Data are plotted as individual values with mean. *P = 0.04 and **P < 0.0001 as compared to CEA10 of respective organs by one-way ANOVA with Dunnett’s multiple comparison.

The primary goal of developing a model of CA is to elucidate the mechanisms that *A. fumigatus* uses to gain access to and grow within the brain to improve our understanding and management of these devastating infections. Previous studies of IPA have demonstrated a role for the pH-responsive *A. fumigatus* transcription factor, PacC, in the adherence and invasion of lung epithelial cells (48). Similarly, the PacC ortholog, Rim101, in *Candida albicans* is required for invasion and damage to host endothelial and epithelial cells (49, 50). Thus, we hypothesized that PacC could be an important factor for interaction with endothelial cells at the BBB to facilitate entry into the brain. To test this, we inoculated A/J mice with WT (CEA10) and two independently isolated PacC deletion mutants (Δ*pacC-1 and* Δ*pacC-2*) and measured the fungal burden at 48 hpi. Consistent with our hypothesis, we observed brain burden in 3/5 mice (~3 × 10^5^ CE; Fig. 4) inoculated with WT, while for each Δ*pacC* mutant, only 1/6 mice had detectable brain burden (~1 × 10^5^ CE; Fig. 4A) at 48 hpi. We also observed a similar trend in the kidneys where all WT inoculated mice had detectable kidney burden (~2 × 10^6^ CE), while only one and two mice had a burden in the Δ*pacC-1* and Δ*pacC-2* inoculated mice, respectively (Fig. 4). We did not observe lung burden in any animals in this experiment. Together, these results suggest that PacC is required for the hematogenous dissemination of *A. fumigatus* to extrapulmonary organs and for entry into the brain to establish CA.

## DISCUSSION

Here, we present a disseminated model of invasive aspergillosis that results in reproducible fungal burden in the brain to study the pathogenesis of CA. Our model uses tail vein inoculation of *A. fumigatus* conidia in C5-complement-deficient mice to establish robust brain burden accompanied by immune cell infiltration, increased vascular leakage, and physical symptoms of CNS disease. Prior to this work, a few models of CA have been developed, some of which primarily use dissemination via tail vein inoculation, some utilize direct inoculation into the brain by intracranial injection, and the recent “two-hit” model, which uses both routes of inoculation (11, 51–53). The advantage of the direct intracranial inoculation model is the establishment of infection specifically in the brain without the high fungal burden in other organs, as often observed in disseminated models including ours presented here. However, the direct injection of *A. fumigatus* conidia into the brain bypasses the interaction with the brain endothelium, a critical step in the initiation of CA from hematogenous dissemination. This inoculation route also introduces local inflammation at the injection site, which may alter the kinetics, magnitude, and type of immune response to *A. fumigatus* conidia.

Models that utilize tail vein inoculation of *A. fumigatus* conidia are often critiqued for being an unnatural route of infection (15) since there is little to no evidence of the presence of conidia after the establishment of invasive disease. We recognize that every animal model has its limitations; however, the utility of being able to study an important and generally unexplored area of fungal pathogenesis outweighs the disadvantages. Fully dissecting the mechanisms of pathogenesis of CA will require the use of several *in vivo* and *in vitro* models, especially when considering the breadth of underlying risk factors and host conditions that render one susceptible to disease. Our goal with this model is to identify pathways and proteins that are essential for pathogenesis in the brain, then validate and further probe these findings with existing *in vitro* and *in vivo* models of invasive aspergillosis and host cell interactions. For example, comparative studies between the pathogenesis of mutants in our disseminated CA model and their pathogenesis in a direct intracranial inoculation model of CA can help us determine whether strains that are defective in establishing disease in the brain are defective in endothelial cell interactions and gaining entry to the brain or growing within the brain tissue itself. Moreover, as we explore this model, we plan to utilize a variety of *A. fumigatus* strains, including those isolated from cerebral infections, to determine whether there are common fungal features that promote brain infection. However, for this work, we focused on the reference strain CEA10, given the wealth of genetic mutants, including mutant libraries that have been generated and characterized in strain background.

While our inflammatory code set is not specific for neuroinflammation, we did observe some significant changes in genes that might represent CNS-specific function. The colony-stimulating factor, CSF1, is a critical regulator of microglial populations and is often upregulated in response to neuroinflammation and damage (54, 55). The activation of microglia by CSF1 results in an M2-like phenotype, which is characterized by other M2 markers such as ARG1 and MRC1, the latter of which is significantly upregulated in response to *A. fumigatus* challenge in the brain (Table 1). These results potentially indicate the presence of an M2 population of microglial cells in CA, though defining the exact populations of immune cells remains an area of future study in this model. We also observed hallmarks of fungal CNS disease that have been observed in previous studies. For example, the production of IL-1β and CXCL1 by microglia is critical in recruiting neutrophils to the brain in response to *Candida albicans* infection (56). Though not statistically significant, we observed the upregulation of chemokines *Cxcl1*, *Cxcl9*, and *Cxcl10* (Table S1) in mice inoculated with *A. fumigatus* versus PBS-inoculated mice, which had no detectable chemokine expression. These chemokines have also been implicated in the response to pulmonary aspergillosis (36, 57). Unfortunately, the murine inflammation code set does not include many of the critical signaling pathways in fungal recognition, including c-type lectins such as Dectin-1 (CLEC7a) and the adapter, CARD9, which drives the production of CXCL1 and IL-1β in *C. albicans* CNS disease (56). It is also important to remember that not all immune regulation is transcriptional, such as the release of mature IL-1β, and therefore this pilot study represents just the beginning of our understanding of the immune response to CA in this model.

Furthermore, while complement-deficient mice are not fully immunocompetent, the use of a well-defined genetic mutation allows us to observe the development of CA in a relatively normal, though delayed immune response. An advantage of using the same mouse background as the majority of *Cryptococcus neoformans* studies is the wealth of information that exists about fungal CNS disease in this mouse strain. This provides the ability to compare the response to each fungal pathogen and determine general antifungal CNS responses and those specific to cerebral aspergillosis. Characterization of the immune response over the course of infection will not only provide insight into what host defenses *A. fumigatus* must overcome to successfully establish CA but also might help to define whether there is a specific immune environment that predisposes disseminated infections to invade brain tissue. Finally, understanding what a relatively “normal” response to CA looks like can help inform host-targeted approaches to the treatment of CA such as the use of immune activators/agonists to promote an anti-fungal immunity in the brain.

With an established model, we tested the hypothesis that the pH-responsive transcription factor PacC is important for entry into the brain given its role in regulating interactions with host cells (48). When delivered into the lungs via intranasal installation, the Δ*pacC* mutant fails to invade pulmonary epithelial cells; however, at least at the early timepoint tested, there is no difference in fungal burden between mice inoculated with WT versus Δ*pacC*. Histological analysis from these mice shows that despite a lack of epithelial invasion, the mutant still germinates and grows within the host suggesting that the virulence of this mutant is governed by defects in host-cell interactions and not just a growth defect within the host (48). Conversely, in *Aspergillus nidulans*, the constitutive activation of PacC results in enhanced virulence marked by increased epithelial invasion, highlighting the role of PacC-regulated genes in the invasive nature of *Aspergillus* (58). The reduction in fungal burden in both the brains and kidneys of mice inoculated with Δ*pacC* suggests this transcription factor is important in regulating endothelial interactions at multiple organs and is not specific to the brain.

Work is ongoing to fully evaluate the role of PacC in CA and to identify other regulators that are critical for endothelial interactions in the brain. However, the model presented here will act as a solid foundation for these studies and will allow us to begin to understand the interactions that occur to allow *A. fumigatus* to access and thrive within the privileged niche of the brain. We expect to identify pathways that are important for general hematogenous dissemination, such as PacC. However, we hope to also identify specific pathways and proteins that are defective only in establishing brain infection, which will highlight brain-specific interactions, though it is possible that general endothelial cell interactions govern dissemination to all extrapulmonary organs. Finally, we will also identify pathways that are essential for growth within the brain tissue itself. Understanding the pathogenesis of CA will allow us to contextualize what we know about *A. fumigatus* in the host and enable comparative studies to identify virulence traits and metabolic pathways that are niche-specific. Ultimately, the goal of these studies is to better understand the pathogenesis of CA and identify novel targets and approaches for the development of more efficacious treatments for those suffering from these deadly and debilitating infections.

## MATERIALS AND METHODS

### Strains, media, and reagents

All *A. fumigatus* strains were maintained on glucose minimal media (59) from 25% glycerol stocks stored at −80°C. *A. fumigatus* CEA10 and CEA17 were received from Dr. Robert Cramer (Dartmouth College), and ATCC13073 (NIH B5233) was purchased from ATCC. Inocula for animal studies were prepared in 0.0001% PBST (PBS [Gibco, Cat# 14190] with Tween-80 [RPI, Cat# P20390]).

### Corticosteroid murine model

Outbred female CD-1 (Envigo) females were treated with a single 40 mg of triamcinolone/kg of body weight (Kenalog-10, Bristol-Myers Squibb) by subcutaneous injection. Twenty-four hours post-treatment, mice were inoculated with 1 × 10^5^ CEA10 conidia in 200 µL PBST via lateral tail vein.

### Complement-deficient murine model

Female A/J (Jackson Labs) mice were inoculated via lateral tail vein with *A. fumigatus* conidia at indicated doses in 200 µL PBST. For intranasal inoculation, mice were anesthetized with isoflurane and inoculated with 5 × 10^6^ ATCC 13073 conidia in 50 µL PBS via intranasal instillation.

### Fungal burden analysis and histology

Lungs, brains, and kidneys were harvested from mice sacrificed 24-, 48-, or 72-hours post-inoculation. Tissues were freeze-dried and homogenized with 2.3 mm zirconia/silica beads with a Fast-Prep24 benchtop bead mill (MP Biomedicals). Tissue was resuspended in TENS buffer (10 mM Tris-HCl pH 7, 150 mM NaCl, 50 mM EDTA, and 0.5% SDS) and incubated for 1 hour at 65°C. Total DNA was isolated with two rounds of phenol:chloroform:IAA extraction followed by one round of chloroform extraction and isopropanol precipitation. A quantity of 500 ng of RNase-treated DNA was used for quantitative PCR to amplify the 18S region using primers SP228 and SP229 (Table S2) and TaqMan probe (/56-FAM/AGCCAGCGGCCCGCAAATG/3IABLFQ/) as previously described (12). Reactions were run using BioRad iQ Supermix (Cat# 1708860) on a BioRad CFX Maestro real-time PCR system. Data are presented as conidial equivalents (genome copies) calculated using the equation: CE = (*N*/6.022 × 10^23^)/(*L* × 10^9^ × 650), where *N* = ng of fungal DNA and *L* = length of DNA template (29 Mb, *A. fumigatus* genome size). All statistical analyses were performed with GraphPad Prism 9. %CV values were calculated using “0” value for all samples below the limit of detection.

For histology, brains from A/J mice were harvested 72 hpi with PBS or 1 × 10^5^ CEA10. Tissues were immersion fixed in 10% buffered formalin, then coronal sections were prepared and embedded for sectioning. Sections were stained with Grogott’s methanamine silver or Hematoxylin and eosin. Images were acquired on a Nikon epifluorescence microscope with a Nikon DS-Fi1 camera and Nikon Elements image acquisition and analysis software. Images were processed with linear brightness and contrast adjustments in Photoshop only to increase ease of viewing.

### Generation of PacC deletion

Strains were generated using CRISPR-Cas9-mediated transformation as previously described (60). The PacC deletion construct was generated using PCR with primers SP230 + SP231 (Table S2), which contain microhomology to the PacC gene flanks, from plasmid pSD38.1 (61) to amplify the *Aspergillus parasitacus* PyrG gene. Protoplasts of CEA17 were generated using VinoTaste Pro (Novozymes) and then transformed with 5 µg of construct, 33 µM RNA duplex solution of each guide (Table S2), and 1 µg/µL Cas9 (IDT, Cat# 1074182). Transformants were plated on a selective media without uridine or uracil. Correct integration of the construct was verified by PCR and then positive transformants were characterized for growth under various pH conditions to verify known phenotypes.

### NanoString analysis

Brains were harvested from female A/J mice inoculated intravenously (as described above) with 1 × 10^5^ *A. fumigatus* CEA10 or PBS at 72 hours post-inoculation. Tissues were freeze-dried and homogenized with 2.3 mm zirconia/silica beads with a Fast-Prep24 benchtop bead mill (MP Biomedicals). RNA was extracted with TriZol reagent and then eluted using gDNA removal and RNeasy columns (Qiagen, Cat# 74134). A quantity of 100 ng of RNA was hybridized with the MMV2 Mouse Inflammation code set probes (NanoString, Cat# XT-CSO-MIN2-12) for 18 hours at 65°C and then run on a NanoString nCounter Pro Analysis System according to the manufacturer’s instructions. Data from Reporter Code Count files were extracted with nSolver software (version 4.0), and then raw counts were exported to Microsoft Excel. Internal negative controls were used to subtract background from raw counts (negative control average + 2 SDs). Counts were normalized across samples by total RNA counts. The probes below the background were set to a value of 1. Data are normalized to the total counts of each sample. Significance was determined using unpaired, two-tailed *t*-tests of control (PBS) mice compared to *A. fumigatus*-inoculated mice.

## SUPPLEMENTAL MATERIAL

Supplemental figures msphere.00468-23-s0001.docx Fig. S1, S2, and S3.

Table S1 msphere.00468-23-s0002.xlsx Expression of all detected genes in NanoString codeset.

Table S2 msphere.00468-23-s0003.docx Primers and guides used in this study.

ASM does not own the copyrights to Supplemental Material that may be linked to, or accessed through, an article. The authors have granted ASM a non-exclusive, world-wide license to publish the Supplemental Material files. Please contact the corresponding author directly for reuse.
