# Supplementary material for: Development of a murine model to study the cerebral pathogenesis of Aspergillus fumigatus
Source: mSphere. 2023 Nov 27;8(6):e00468-23. doi: 10.1128/msphere.00468-23 (PMC10732035; doi:10.1128/msphere.00468-23)
Supplement: Supplemental figures — Fig. S1, S2, and S3. [file msphere.00468-23-s0001.docx]

**
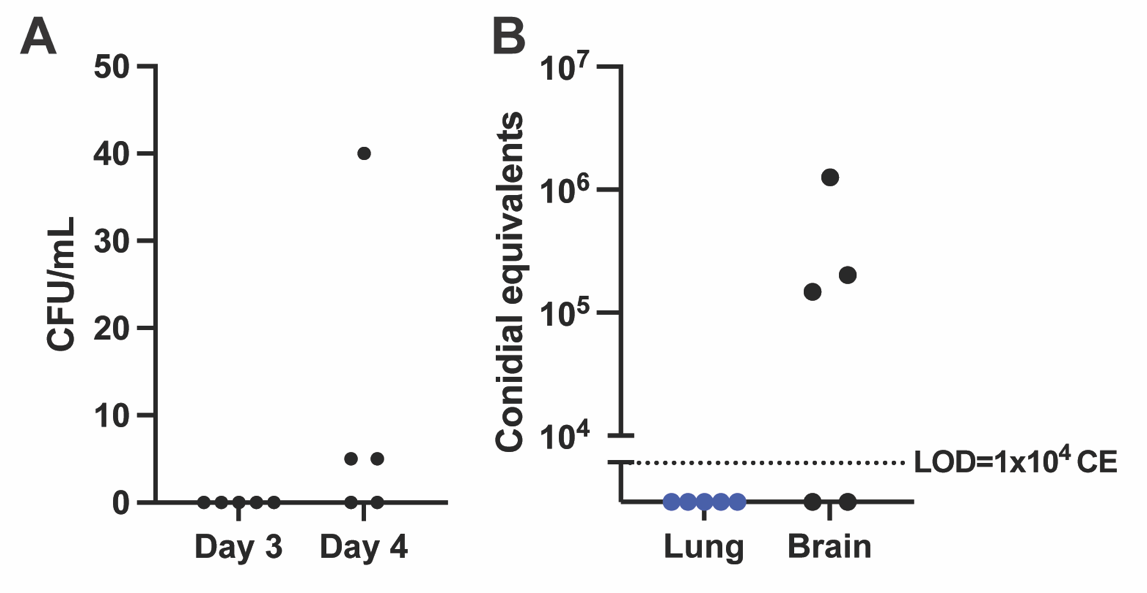
Figure S1. Outbred mice do not develop robust cerebral infection following intranasal instillation in corticosteroid treated mice or intravenous inoculation of *A. fumigatus* conidia without immune suppression**. A) Fungal burden of brains harvested at 3- and 4- days post inoculation from Kenalog-treated CD-1 mice inoculated with 2x10^6^ ATCC13073 conidia via intranasal instillation. Brains were homogenized then undiluted homogenate was plated on YPD and colonies were enumerated after 48 hours growth at 37ºC. B) Fungal burden of lungs harvested 3 days post inoculation and brains of immune competent CD-1 mice inoculated with 1x10^6^ ATCC13073 conidia via lateral tail vein. Burden is shown as conidial equivalents as measured by qRT-PCR of DNA extracted from whole organs. Dashed line represents limit of detection (LOD); n=5 mice per group.


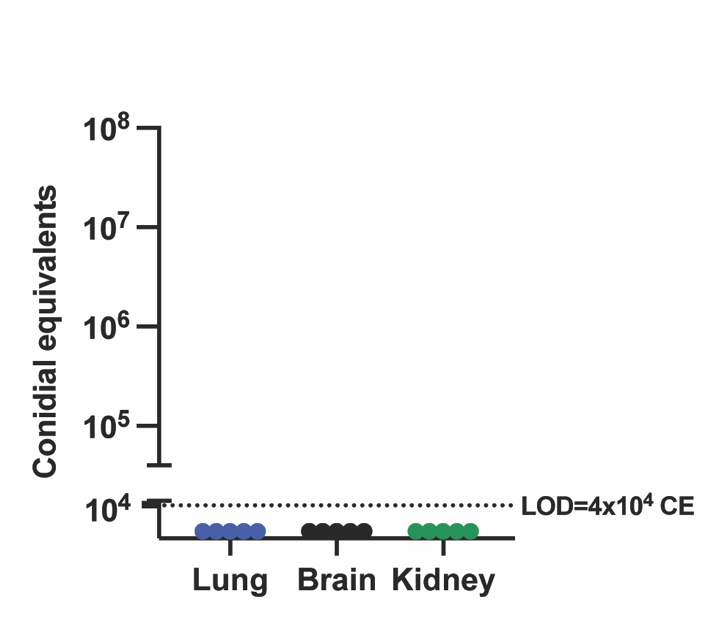


**Figure S2**. Fungal burden of mice inoculated with 1x10^5^ CEA17 (uracil/uridine auxotroph) conidia at 72 hours post inoculation. Dotted line represents the limit of detection (LOD) for fungal burden analyses. Data are plotted as individual values with mean.


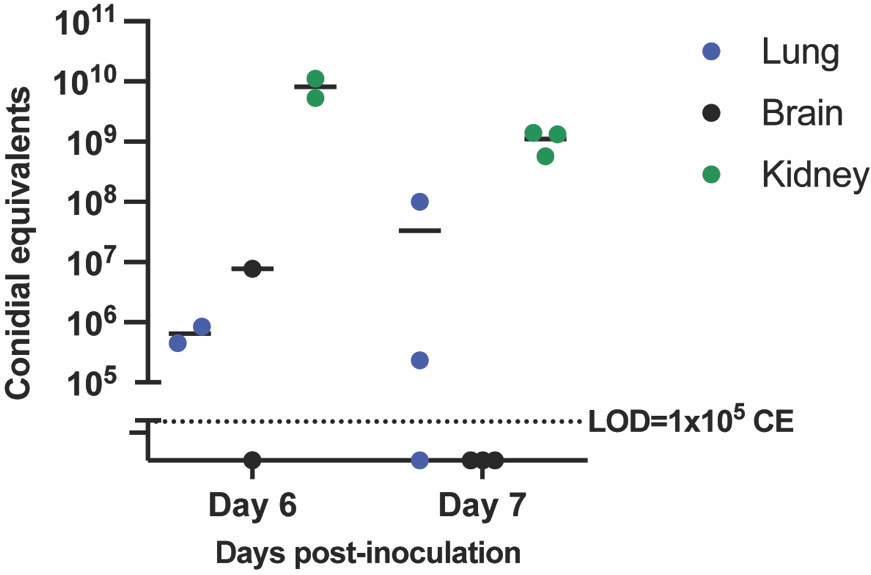


**Figure S3. A/J mice do not develop robust brain burden with a low dose inoculum.** Fungal burden at the time of euthanasia of A/J mice inoculated with 5x10^4^ CEA10 conidia via lateral tail vein. n=2 mice at day 6 and 3 mice at day 7. Dashed line represents limit of detection (LOD). Data are plotted as individual values with mean.
