# Supplementary material for: Development of a murine model to study the cerebral pathogenesis of Aspergillus fumigatus
Source: mSphere. 2023 Nov 27;8(6):e00468-23. doi: 10.1128/msphere.00468-23 (PMC10732035; doi:10.1128/msphere.00468-23)
Supplement: Table S2 — Primers and guides used in this study. [file msphere.00468-23-s0003.docx]

| **Name** | **Description** | **Sequence** |
| --- | --- | --- |
| SP228 | Fungal burden Fw | GGCCCTTAAATAGCCCGGT |
| SP229 | Fungal burden Rv | TGAGCCGATAGTCCCCCTA |
| SP230 | 5' PacC microhomology | GCCAATGTCGATCCAGCCGTGTCATTTCCCTTTCTACGG  AGTTGACCGGGaccggtcgcctcaaacaatgctct |
| SP231 | 3' PacC microhomology | CCCGGTCAACTCCGTAGAAAGGGAAATGACACGGCTGG  ATCGACATTGGCgtctgagaggaggcactgatgcg |
| PacC gRNA 1 | PacC guide RNA | GCAGCCTGCAGCTACAACCC |
| PacC gRNA 2 | PacC guide RNA | ATTGCATCCCGTCTGGAAAG |

Table S2. Primers and guides used in this study.
